# Possible Involvement of Nitric Oxide and Reactive Oxygen Species in Glucose Deprivation-Induced Activation of Transcription Factor Rst2

**DOI:** 10.1371/journal.pone.0078012

**Published:** 2013-10-14

**Authors:** Toshiaki Kato, Xin Zhou, Yan Ma

**Affiliations:** 1 Division of Molecular Pharmacology and Pharmacogenomics, Department of Biochemistry and Molecular Biology, Kobe University Graduate School of Medicine, Kobe, Japan; 2 The First Affiliated Hospital of Liaoning Medical University, Jinzhou City, Liaoning Province, China; Kinki University School of Pharmaceutical Sciences, Japan

## Abstract

Glucose is one of the most important sources of cellular nutrition and glucose deprivation induces various cellular responses. In *Schizosaccharomyces pombe*, zinc finger protein Rst2 is activated upon glucose deprivation, and regulates gene expression via the STREP (stress response element of *Schizosaccharomyces pombe*) motif. However, the activation mechanism of Rst2 is not fully understood. We monitored Rst2 transcriptional activity in living cells using a *Renilla* luciferase reporter system. Hydrogen peroxide (H_2_O_2_) enhanced Rst2 transcriptional activity upon glucose deprivation and free radical scavenger inhibited Rst2 transcriptional activity upon glucose deprivation. In addition, deletion of the *trx2* ^*+*^ gene encoding mitochondrial thioredoxin enhanced Rst2 transcriptional activity. Notably, nitric oxide (NO) generators enhanced Rst2 transcriptional activity upon glucose deprivation as well as under glucose-rich conditions. Furthermore, NO specific scavenger inhibited Rst2 transcriptional activity upon glucose deprivation. Altogether, our data suggest that NO and reactive oxygen species may be involved in the activation of transcription factor Rst2.

## Introduction

Glucose is the main source of energy for most cells and glucose deprivation induces various cellular processes including gene expression, metabolic change, and oxidative stress [1-3]. The fission yeast *Schizosaccharomyces pombe* (*S. pombe*) is a good model system for studying mechanisms of glucose deprivation-induced gene expression in higher eukaryotes [4].

Zinc-finger protein Rst2 plays an important role in glucose deprivation-induced gene expression. Upon glucose deprivation, Rst2 induced expression of the *fbp1* ^+^ gene, encoding a fructose-1,6-bis-phosphatase, via the STREP (stress response element of *Schizosaccharomyces pombe*) motif [5]. It has also been demonstrated that under glucose-rich conditions, cAMP-dependent kinase (PKA) directly phosphorylates and inhibits Rst2. Upon glucose deprivation PKA-independent activation of Rst2 is observed [5], however, the mechanism is not well understood.

In our previous study, we developed a method to monitor the transcriptional activity in living cells [6]. To identify the activation mechanisms of Rst2, we monitored Rst2 transcriptional activity. The results show that hydrogen peroxide (H_2_O_2_) and nitric oxide (NO) generators enhanced Rst2 transcriptional activity. Free radical scavenger and NO specific scavenger inhibited glucose deprivation-induced activation of Rst2. These results highlight that reactive oxygen species (ROS) and NO may be involved in the activation of Rst2.

## Materials and Methods

### Strains, Media, and Genetic and Molecular Biology Methods

*S. pombe* strains used in this study are listed in Table 1. The normal minimal medium EMM (Edinburgh minimal medium), low glucose EMM and YES media have been described previously [7-9]. Standard genetic and recombinant-DNA methods [10] were used except where noted.

**Table 1:** Strains used in this study.

Strain	Genotype	Reference
HM123	*h^-^ leu1-32*	Our stock
KP133	*h* ^-^ *leu1-32 ura4*-*D18 pap1*::*ura4* ^+^	[38]
KP471	*h* ^-^ *leu1-32 ura4-D18 sty1*::*ura4* ^+^	[8]
KP2637	*h* ^-^ *leu1-32 ura4-D18 ade6-M210 rst2*::*ura4* ^+^	[5]
KP2691	*h* ^-^ *leu1-32 ura4-D18 rst2*::*ura4* ^+^	This study
KP2921	*h* ^-^ *leu1-32 ura4-D18 pka1*::*ura4* ^+^	[39]
KP2945	*h* ^+^ *ade6-M210 tpx1*::*ura4* ^+^ *his7-366 ura4-D18*	[40]
KP3015	*h* ^-^ *leu1-32 ura4-D18 tpx1*::*ura4* ^+^	This study
KP3157	*h* ^*-*^ * leu1-32 lys3*:: *loxp*	[11]
KP5180	*h* ^*-*^ * leu1-32 trx2*:: *KanMX*_*4*_	This study
KP5383	*h* ^-^ *leu1-32 lys3*::*loxp trx1*::*lys3* ^+^	This study
KP92765	*h* ^-^ *ade6-M210 ura4-D18 leu1-32 trx2*::*KanMX*_*4*_	[41]

### Disruption of the *trx1* ^*+*^ Gene

To knockout the *trx1* ^+^gene, a PCR-based targeted gene deletion method was prepared by the Cre-loxP-mediated marker removal procedure as described previously [11]. The DNA fragments containing the disrupted *trx1* ^+^, were amplified by using the plasmid pKB6640 which contains the *lys3* ^+^ marker as a template [11], and using the sense primer 5′-cgt taa atc gat ttt ttc ttt att tga gta tat att ttt aac tta att tcc cat ttc att tat ata caa cCC AAT AGG CCG AAA TCG GCA AAA TCC C-3′, and the antisense primer 5′-cat tta ttt ttg tta aat aaa aat att ttg tat tac aag ttc ata aca act aac tat cag att gcg taa aGG TGA TGG TTC ACG TAG TGG GCC-3′. The resulting products containing *trx1*::*lys3* ^+^ disruption fragments were transformed into KP3157 (*h* ^-^ *leu1-32 lys3*::*loxp*) cells [11]. Stable integrants were selected on medium lacking lysine. The disruption of the gene was checked using PCR (data not shown).

### Construction of Reporter Plasmid

The 3xCRE sequence of pKB5878 (3xCRE::*Renilla*) [6] was replaced with 3xSTREP sequence using oligonucleotides (sense: 5′-GGC TTC CCC TCA TAC ACC CCT CAT ACA CAC CCC TCA TGC AC-3′, antisense: 5′-TCG AGT GCA TGA GGG GTG TGT ATG AGG GGT GTA TGA GGG GAA GCC TGC A-3′, STREP sequence underlined), to give pKB8307 (3xSTREP::*Renilla*).

### Real-Time Monitoring Assay of Rst2-Mediated Transcriptional Activity

The multi-copy reporter plasmid (pKB8307) was transformed into fission yeast cells for reporter assays. The transformants were cultured at 27°C in normal EMM media overnight to midlog phase and recovered by centrifugation. Then the cells were resuspended in refresh EMM containing 2% glucose as glucose-rich medium (GR), or in low glucose EMM containing 0.1% glucose to induce glucose deprivation (GD). Coelenterazine was used as a substrate for Renilla luciferase and yielding luminescence was detected using a luminometer (AB-2350; ATTO Co., Tokyo, Japan) at 1-min intervals and reported as relative light units (RLU).

## Results

### Real-Time Monitoring of Rst2 Transcriptional Activity in Living Cells

**Figure 1:**
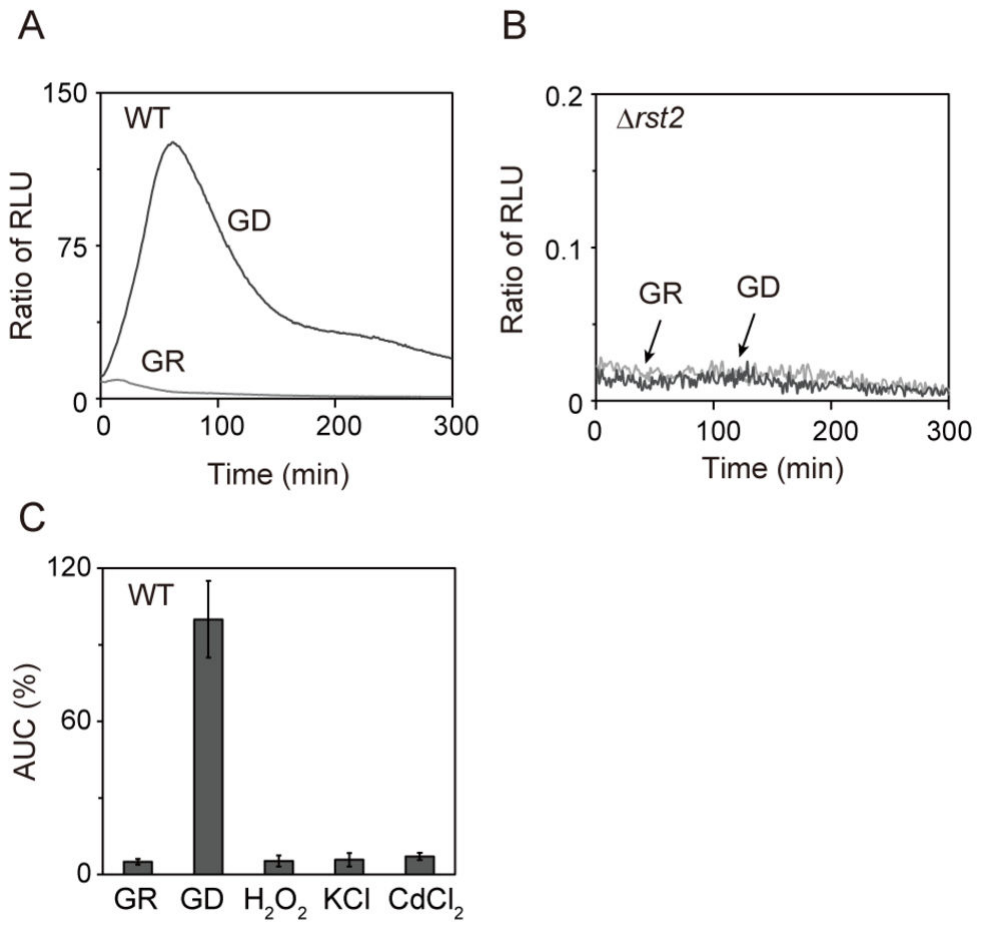
Monitoring of Rst2 transcriptional activity in living cells by using the *Renilla* luciferase reporter assay. (A) Glucose deprivation induced a marked increase in transcriptional activation. Wild-type cells harboring the reporter plasmid were cultured and assayed as described under “Materials and Methods”. GR (light gray line) indicates that the cells were resuspended in glucose-rich medium (GR). GD (dark gray line) indicates that the cells were resuspended in low glucose medium to induce glucose deprivation (GD). Y-axis values are the ratio of relative light units (RLU) of each sample to that of wild-type cells in GR at 150 minutes. The data shown are representative of multiple experiments. (B) Glucose deprivation-induced transcriptional activation is completely abolished in Δ*rst2* cells. The Δ*rst2* cells harboring the reporter plasmid were cultured and assayed as described in Figure 1A. (C) Rst2 is specifically activated by glucose deprivation. Wild-type cells harboring the reporter plasmid were treated with GR, GD, 1 mM H_2_O_2_, 300 mM KCl or 1 mM CdCl_2_ as indicated. Area under the curve (AUC) is expressed as a percentage of RLU of wild-type cells in GD from 0 to 300 minutes. Error bars, mean ± S.D. (n ≥ 3).

Transcriptional factor Rst2 regulates gene expression via the STREP motif [5]. We constructed reporter plasmid containing three tandem repeats of STREP fused to *Renilla* luciferase (3xSTREP::*Renilla*). In wild-type cells, glucose deprivation caused a marked increase in the transcription with a peak at about 80 min (Figure 1A). In Δ*rst2* cells, glucose deprivation-induced transcription was completely abolished (Figure 1B). These results indicate that the reporter assay reflects Rst2 transcriptional activity.

Previous work indicated that Rst2 is activated by glucose deprivation [5]. To examine whether Rst2 is specifically activated by glucose deprivation, wild-type cells were subjected to oxidative stress (1 mM H_2_O_2_), osmotic stress (300 mM KCl) or heavy metal stress (1 mM CdCl_2_), respectively. The results clearly showed that 3xSTREP::*Renilla* responded to glucose deprivation, but not H_2_O_2_, KCl or CdCl_2_ (Figure 1C).

### PKA Inhibited Rst2 Transcriptional Activity

**Figure 2:**
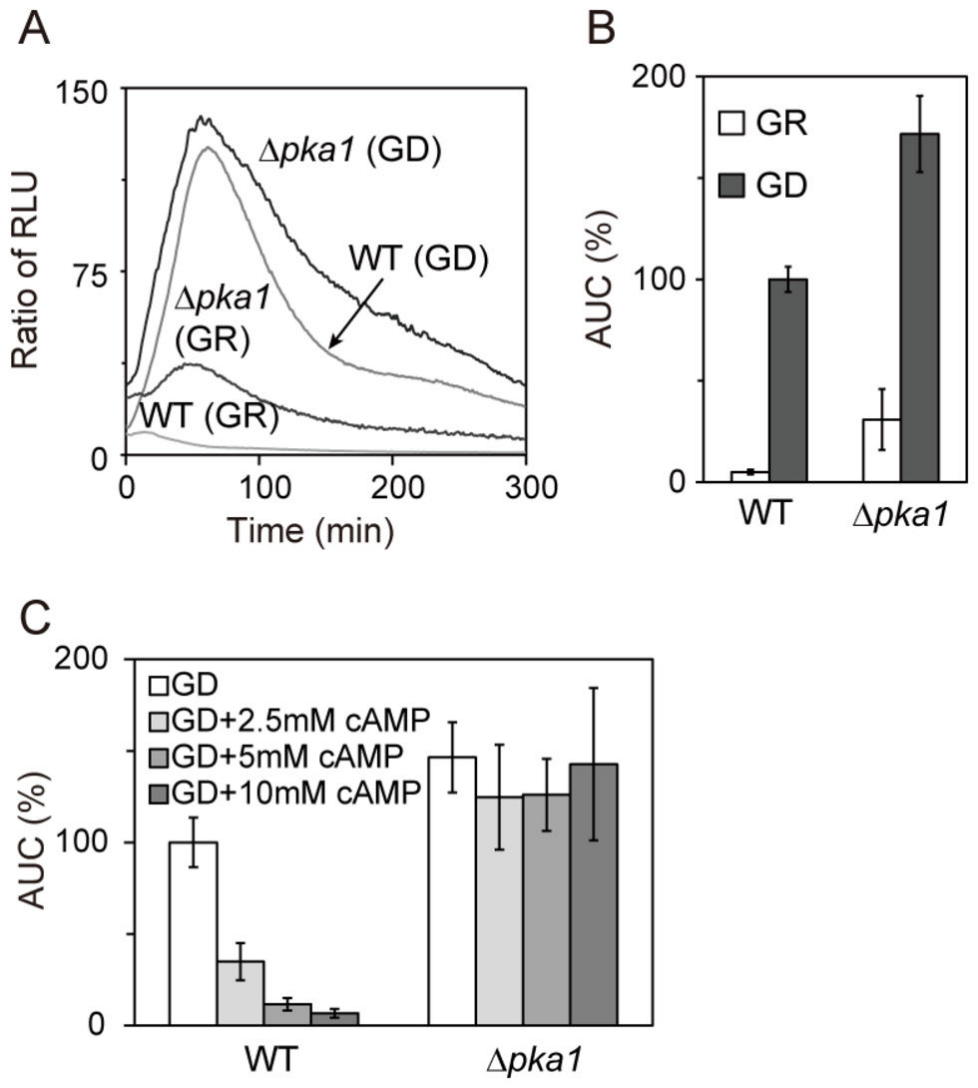
PKA inhibited Rst2 transcriptional activity. (A and B) Deletion of the *pka1* ^+^ gene enhanced Rst2 transcriptional activity. The wild-type and Δ*pka1* cells harboring the reporter plasmid were cultured and assayed as described in Figure 1A. (C) The effect of cAMP on Rst2 transcriptional activity. The wild-type and Δ*pka1* cells harboring the reporter plasmid were treated with GD in the presence or absence cAMP (2.5 mM to 10 mM). Error bars, mean ± S.D. (n ≥ 3).

*S. pombe* has a single gene encoding the catalytic subunit of PKA, *pka1* ^+^ [12]. Previous work indicated that Rst2 is phosphorylated and inhibited by PKA under glucose-rich conditions [5]. We then monitored Rst2 transcriptional activity in Δ*pka1* cells. The Δ*pka1* cells showed high basal transcription activity with normal response to glucose deprivation (Figure 2A and B). We also monitored whether glucose deprivation-induced activation of Rst2 is repressed by adenosine-3′,5′-cyclic monophosphate (cAMP) addition. In wild-type cells, the addition of cAMP caused a dose-dependent decrease in glucose deprivation-induced activation of Rst2, whereas cAMP did not significantly inhibit Rst2 transcriptional activity in Δ*pka1* cells (Figure 2C). The results indicate that cAMP inhibited glucose deprivation-induced activation of Rst2 through PKA. Altogether, these results suggest that PKA functions as a negative regulator of Rst2 and other mechanisms may be involved in the activation of Rst2.

### Redox Change May Be Involved in Glucose Deprivation-Induced Transcriptional Activation of Rst2

**Figure 3:**
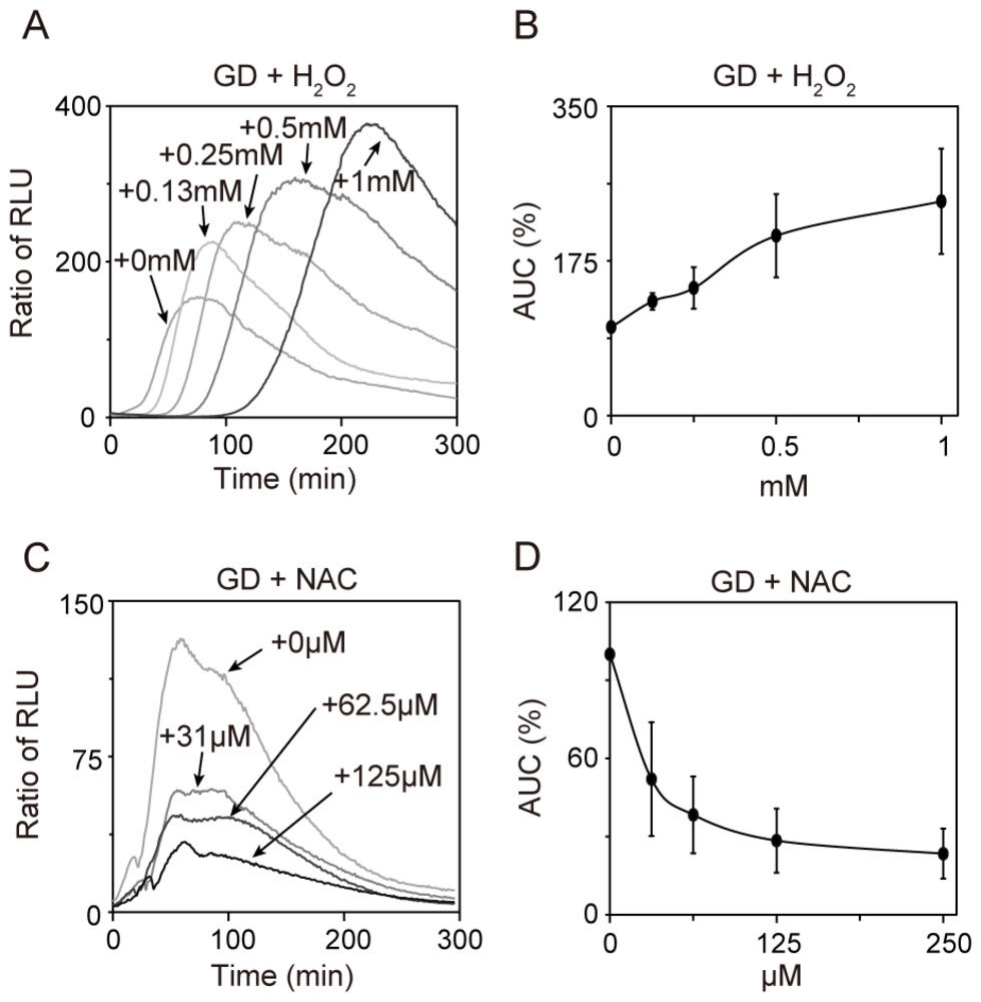
Redox changes affected Rst2 transcriptional activity upon glucose deprivation. (A and B) H_2_O_2_ enhanced Rst2 transcriptional activity upon glucose deprivation. Wild-type cells harboring the reporter plasmid were treated with GD in the presence or absence of H_2_O_2_ (0.125 mM to 1.0 mM). (C and D) NAC inhibited Rst2 transcriptional activity upon glucose deprivation. Wild-type cells harboring the reporter plasmid were treated with GD in the presence or absence of NAC (31 μM to 250 μM).

Free radical ROS, such as H_2_O_2_ and superoxide, cause oxidative stress and act as signal molecules [13]. Previous work indicated that glucose deprivation induces oxidative stress in *S. pombe* [14]. These results led us to investigate the relationship between free radical ROS and Rst2 transcriptional activity. Under glucose-rich conditions, 1 mM H_2_O_2_ did not affect Rst2 transcriptional activity (Figure 1C). In contrast, H_2_O_2_ caused a dose-dependent increase in Rst2 transcription activity upon glucose deprivation (Figure 3A and B). Free radical scavenger N-acetyl-L-cysteine (NAC; NACALAITESQUE, INC.) inhibits the oxidative stress-induced activation of the Sty1 MAPK pathway [6]. We next addressed whether NAC inhibits glucose deprivation-induced activation of Rst2. NAC caused a dose-dependent decrease in glucose deprivation-induced activation of Rst2 (Figure 3C and D). These results suggest that free radical ROS may be involved in the Rst2 transcriptional activation induced by glucose deprivation.

We previously demonstrated that H_2_O_2_ activates Sty1 and that NAC inhibits oxidative stress-induced activation of Sty1 [6]. In Δ*sty1* cells, H_2_O_2_ increased Rst2 transcriptional activity upon glucose deprivation, and NAC inhibited Rst2 transcriptional activity upon glucose deprivation (data not shown). The results indicate that the effect of H_2_O_2_ or NAC on Rst2 activity is independent on Sty1.

### Deletion of the *trx2* ^+^ Gene Enhanced Rst2 Transcriptional Activity

**Figure 4:**
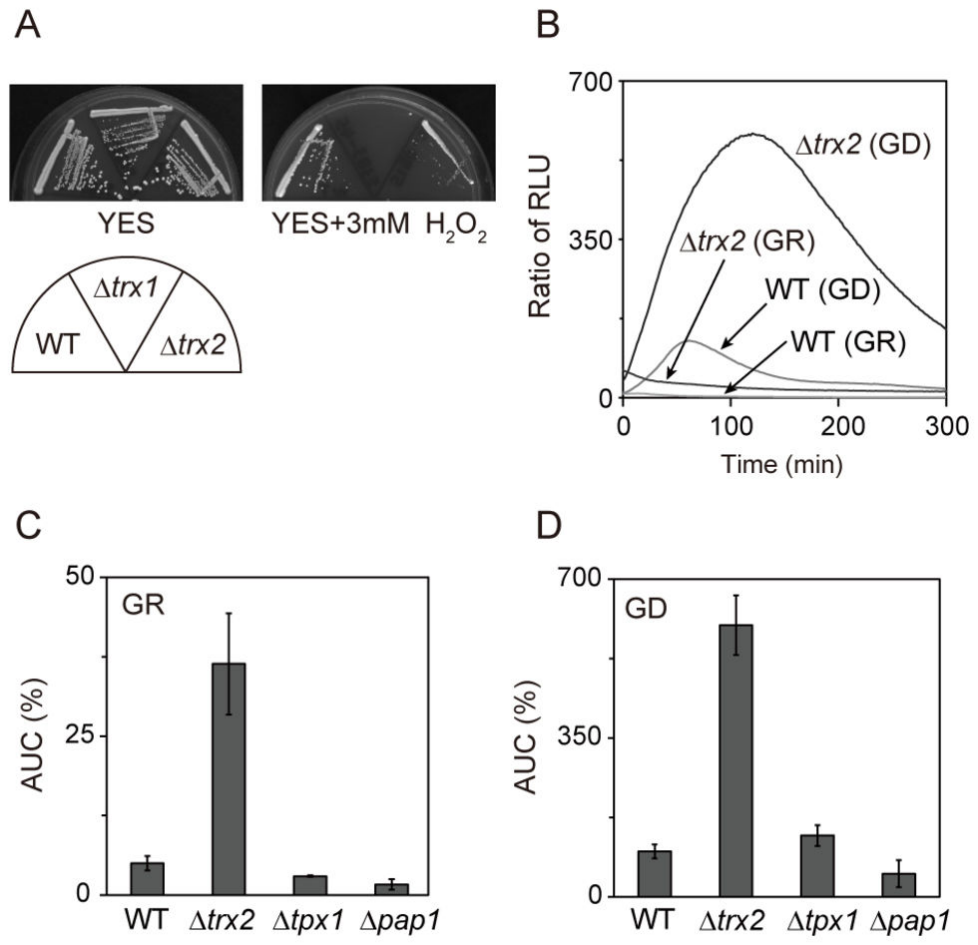
Deletion of the *trx2* ^+^ gene enhanced Rst2 transcriptional activity. (A) The Δ*trx1* and Δ*trx2* cells showed H_2_O_2_-sensitive phenotype. Wild-type, Δ*trx1* and Δ*trx2* cells were streaked onto YES plates with or without 3 mM H_2_O_2_, and cultured at 30°C for 3 days. (B) Deletion of the *trx2* ^+^ gene enhanced Rst2 transcriptional activity. Wild-type and Δ*trx2* cells harboring the reporter plasmid were cultured and assayed as described in Figure 1A. (C and D) Deletion of the *trx2* ^+^ gene specifically enhanced Rst2 transcriptional activity. Wild-type, Δ*trx2*, Δ*tpx1* and Δ*pap1* cells harboring the reporter plasmid were cultured and assayed as described in Figure 1A. Error bars, mean ± S.D. (n ≥ 3).

The free radical scavenger thioredoxin is conserved from prokaryote to eukaryote and plays a role in maintaining the cellular redox environment [15]. There are two thioredoxins, cytosolic thioredoxin Trx1 and mitochondrial thioredoxin Trx2 in *S. pombe* [16]. We looked at H_2_O_2_ sensitivity of Δ*trx1* and Δ*trx2* cells. The results showed that on YES containing 3 mM H_2_O_2_ the growth of Δ*trx1* cells was completely inhibited, whereas that of Δ*trx2* cells was partially inhibited (Figure 4A). These results indicate that both cytosolic and mitochondrial thioredoxins are important in the detoxification of H_2_O_2_. It is demonstrated that the Δ*trx1* cells required cysteine for growth [17,18]. Consistently, the Δ*trx1* cells grew as well as wild-type cells on EMM supplemented with 500 mg/l cysteine whereas they failed to grow on EMM without cysteine (Figure S1A).

The cytosolic thioredoxin peroxidase Tpx1 and the transcription factor Pap1 play a role in defense against oxidative stress in *S. pombe* [19,20]. Therefore, we monitored Rst2 transcriptional activity in Δ*trx2*, Δ*pap1*, Δ*tpx1*, and Δ*trx1* cells. In Δ*trx2* cells, Rst2 transcriptional activity was higher than that in wild-type cells under both glucose-rich and glucose-deprived conditions (Figure 4B-D). In Δ*tpx1* and Δ*pap1* cells, Rst2 transcriptional activity was similar to that observed in wild-type cells (Figure 4C and D). Unexpectedly, in Δ*trx1* cells, Rst2 transcriptional activity was lower than that in wild-type cells under both conditions (Figure S1B). These results suggest that intracellular redox state affects Rst2 transcriptional activity.

### NO May Be Involved in the Transcriptional Activation of Rst2

**Figure 5:**
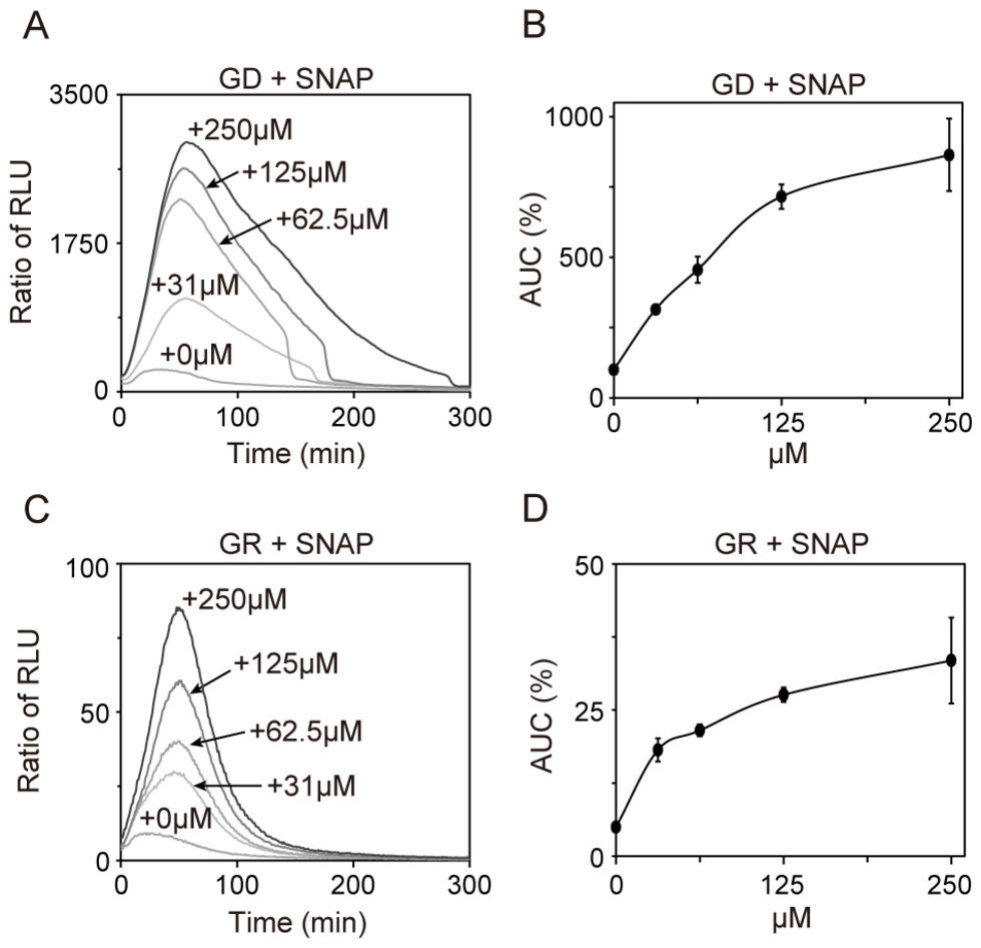
NO generator SNAP activated Rst2 transcriptional activity. (A and B) SNAP enhanced Rst2 transcriptional activity upon glucose deprivation. Wild-type cells harboring the reporter plasmid were assayed in GD media in the presence or absence of SNAP. (C and D) SNAP enhanced Rst2 transcriptional activity under glucose-rich conditions. Wild-type cells harboring the reporter plasmid were assayed in GR media in the presence or absence of SNAP.

**Figure 6:**
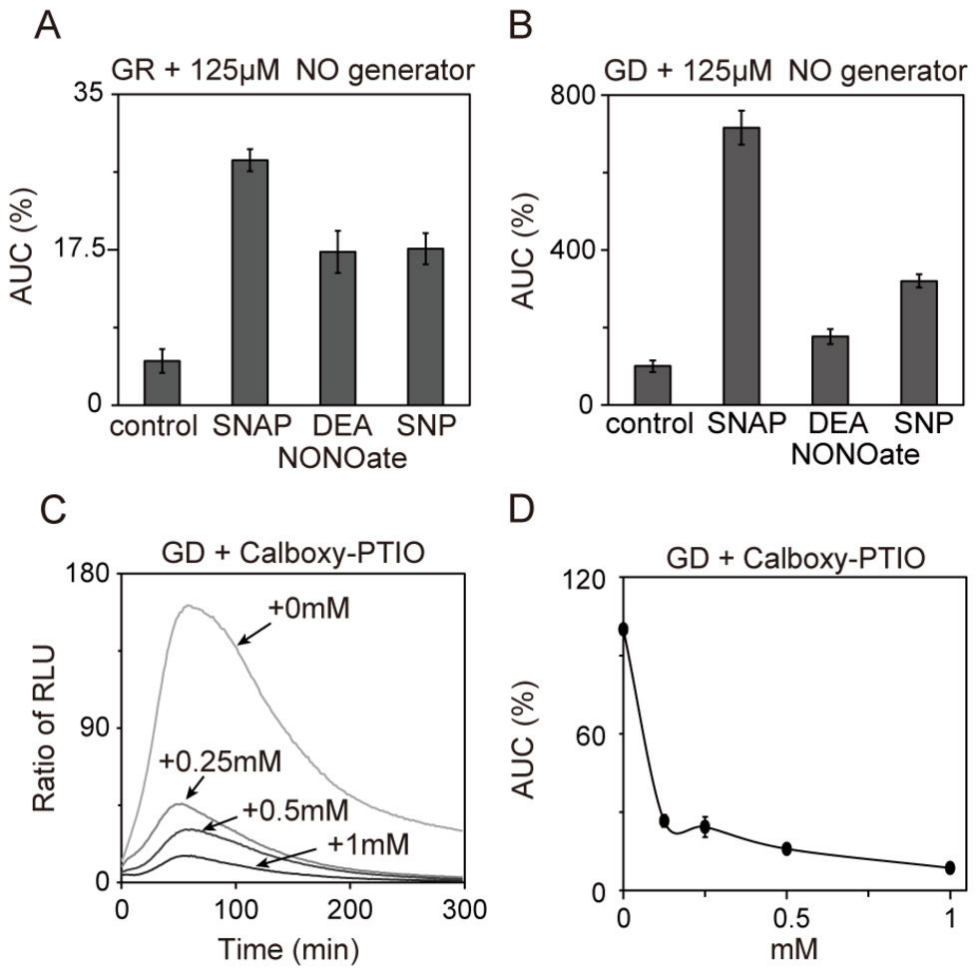
NO may be involved in the activation of Rst2. (A and B) NO generators activated Rst2 transcriptional activity. Wild-type cells harboring the reporter plasmid were treated with GR or GD in the presence or absence of 125 μM NO generators (SNAP, DEA NONOate or SNP). (C and D) Calboxy-PTIO inhibited Rst2 transcriptional activity upon glucose deprivation. Wild-type cells harboring the reporter plasmid were treated with GD in the presence or absence of calboxy-PTIO (0.125 mM to 1 mM). Error bars, mean ± S.D. (n ≥ 3).

Nitric oxide (NO) is also a free radical and acts as a signal molecule [21]. In mammalian cells, NO modulates various cellular processes including gene expression, metabolism, and mitochondrial function [21-23]. In *S. pombe*, NO may function as a signal molecule which induces transcriptional and physiological changes [24]. Here, we examined the effect of the NO generator *S*-Nitroso-*N*-acetylpenicillamine (SNAP; Wako) on Rst2 activation. Results showed that unlike H_2_O_2_, SNAP induced a dose-dependent increase in Rst2 transcriptional activity under both conditions (Figure 5A-D). Similarly, other nitric oxide generators such as sodium nitroprusside dehydrate (SNP; Enzo) and diethylamine-NONOate (DEA-NONOate; Enzo) also increased Rst2 transcriptional activity under both conditions (Figure 6A and B). 

Next, we examined the effect of 2-(4-Carboxyphenyl)-4,4,5,5-tetramethylimidazoline-1-oxyl-3-oxide (carboxy-PTIO; Dojindo), a NO specific scavenger [25] on Rst2 transcriptional activity. The results showed that carboxy-PTIO inhibited glucose deprivation-induced activation of Rst2 in a dose-dependent manner (Figure 6C and D). These results suggest that NO may be involved in the transcriptional activation of Rst2. In addition, we examined whether the effect of NO on Rst2 activity is dependent on PKA. In Δ*pka1* cells, SNAP increased Rst2 transcriptional activity under glucose-rich condition (Figure S2). The result indicates that the effect of NO on Rst2 activity is independent on PKA.

## Discussion

Here we show that free radicals, NO and ROS, caused a dose-dependent increase in Rst2 transcriptional activity upon glucose deprivation. NO specific scavenger carboxy-PTIO and free radical scavenger NAC caused a dose-dependent decrease in glucose deprivation-induced activation of Rst2. These results suggest that NO and/or ROS may be involved in glucose deprivation-induced activation of transcription factor Rst2. We also show that under glucose-rich conditions, NO, but not ROS, induced Rst2 transcriptional activation. Previous work demonstrated that NO and ROS affect cellular responses in part through reversible thiol modifications [23,26-28]. Cross-talk between these reactive species might be common and have potentially important implications for normal and pathological cellular functions [29-32]. Altogether, these results indicate that H_2_O_2_ and NO may act by different mechanisms.

*S*-nitrosylation, the covalent attachment of NO to cysteine thiol, regulates various cellular processes including gene expression and signal transduction [23,33]. We show that SNAP induced a markedly higher Rst2 transcriptional activity compared with DEA NONOate. Consistently, it is known that SNAP is a more potent reagent than DEA NONOate in inducing *S*-nitrosylation [34]. Therefore, we hypothesize that *S*-nitrosylation level may affect Rst2 transcriptional activity.

Thioredoxin has been implicated in the regulation of the redox state of ROS-responsive signaling proteins [35,36]. Glucose deprivation induces mitochondrial ROS generation [2], and the mitochondrial thioredoxin modulates ROS emission from mitochondria [37]. Here, the mitochondrial thioredoxin Trx2 deletion cells showed higher Rst2 transcriptional activity than that in wild-type cells, whereas cytosolic antioxidant enzyme Trx1 or Tpx1, or oxidative stress response transcription factor Pap1 deletion cells did not enhance the activity. We hypothesize that mitochondrial ROS generation enhances Rst2 transcriptional activity. Also, multiple studies reported that thioredoxin may play an important role in protein denitrosylation [29,30]. In combination with our results, we hypothesize that in fission yeast, glucose deprivation induced the generation of NO and/or ROS in mitochondria that in turn resulted in the activation of Rst2.

## Supporting Information

Figure S1 **Monitoring of Rst2 transcriptional activity in Δ*trx1* cells.** (A) Deletion of the trx1^+^ gene caused cysteine auxotrophy. Wild-type and Δ*trx1* cells were streaked onto EMM containing 50 mg/l leucine in the presence (+ Cysteine) or absence of 500 mg/l cysteine (- Cysteine), and cultured at 30°C for 3 days. (B) Monitoring of Rst2 transcriptional activity in Δ*trx1* cells. Wild-type and Δ*trx1* cells harboring the reporter plasmid were treated with GR and GD in the presence of 500 mg/l cysteine. Error bars, mean ± S.D. (n ≥ 3).
(TIF)

Figure S2 **SNAP activated Rst2 transcriptional activity in Δ*pka1* cells.** The Δ*pka1* cells harboring the reporter plasmid were assayed in GR media in the presence or absence of SNAP. Error bars, mean ± S.D. (n ≥ 3).
(TIF)

## References

[1] Vaulont S, Vasseur-Cognet M, Kahn A (2000) Glucose regulation of gene transcription. J Biol Chem 275: 31555-31558. doi:10.1074/jbc.R000016200. PubMed: 10934218. 10934218

[2] Ferretti AC, Larocca MC, Favre C (2012) Nutritional stress in eukaryotic cells: oxidative species and regulation of survival in time of scarceness. Mol Genet Metab 105: 186-192. doi:10.1016/j.ymgme.2011.11.007. PubMed: 22192525. 22192525

[3] Santangelo GM (2006) Glucose signaling in *Saccharomyces* *cerevisiae* . Microbiol Mol Biol Rev 70: 253-282. doi:10.1128/MMBR.70.1.253-282.2006. PubMed: 16524925. 16524925 PMC1393250

[4] Hoffman CS, Winston F (1990) Isolation and characterization of mutants constitutive for expression of the *fbp1* gene of *Schizosaccharomyces* *pombe* . Genetics 124: 807-816. PubMed: 2157626. 2157626 10.1093/genetics/124.4.807 PMC1203973

[5] Higuchi T, Watanabe Y, Yamamoto M (2002) Protein kinase A regulates sexual development and gluconeogenesis through phosphorylation of the Zn finger transcriptional activator Rst2p in fission yeast. Mol Cell Biol 22: 1-11. doi:10.1128/MCB.22.1.1-11.2002. PubMed: 11739717. 11739717 PMC134213

[6] Zhou X, Ma Y, Kato T, Kuno T (2012) A measurable activation of the bZIP transcription factor Atf1 in a fission yeast strain devoid of stress-activated and cell-integrity MAPK activities. J Biol Chem 287: 23434-23439. doi:10.1074/jbc.C111.338715. PubMed: 22661707. 22661707 PMC3390620

[7] Toda T, Dhut S, Superti-Furga G, Gotoh Y, Nishida E et al. (1996) The fission yeast *pmk1* ^+^ gene encodes a novel mitogen-activated protein kinase homolog which regulates cell integrity and functions coordinately with the protein kinase C pathway. Mol Cell Biol 16: 6752-6764. PubMed: 8943330. 8943330 10.1128/mcb.16.12.6752 PMC231678

[8] Zhou X, Ma Y, Sugiura R, Kobayashi D, Suzuki M et al. (2010) MAP kinase kinase kinase (MAPKKK)-dependent and -independent activation of Sty1 stress MAPK in fission yeast. J Biol Chem 285: 32818-32823. doi:10.1074/jbc.M110.135764. PubMed: 20729203. 20729203 PMC2963339

[9] Ryuko S, Ma Y, Ma N, Sakaue M, Kuno T (2012) Genome-wide screen reveals novel mechanisms for regulating cobalt uptake and detoxification in fission yeast. Mol Genet Genomics 287: 651-662. doi:10.1007/s00438-012-0705-9. PubMed: 22806344. 22806344

[10] Moreno S, Klar A, Nurse P (1991) Molecular genetic analysis of fission yeast *Schizosaccharomyces* *pombe* . Methods Enzymol 194: 795-823. doi:10.1016/0076-6879(91)94059-L. PubMed: 2005825. 2005825

[11] Ma Y, Sugiura R, Saito M, Koike A, Sio SO et al. (2007) Six new amino acid-auxotrophic markers for targeted gene integration and disruption in fission yeast. Curr Genet 52: 97-105. doi:10.1007/s00294-007-0142-1. PubMed: 17622533. 17622533

[12] Maeda T, Watanabe Y, Kunitomo H, Yamamoto M (1994) Cloning of the *pka1* gene encoding the catalytic subunit of the cAMP-dependent protein kinase in *Schizosaccharomyces* *pombe* . J Biol Chem 269: 9632-9637. PubMed: 8144551. 8144551

[13] Dröge W (2002) Free radicals in the physiological control of cell function. Physiol Rev 82: 47-95. PubMed: 11773609. 11773609 10.1152/physrev.00018.2001

[14] Madrid M, Soto T, Franco A, Paredes V, Vicente J et al. (2004) A cooperative role for Atf1 and Pap1 in the detoxification of the oxidative stress induced by glucose deprivation in *Schizosaccharomyces* *pombe* . J Biol Chem 279: 41594-41602. doi:10.1074/jbc.M405509200. PubMed: 15247218. 15247218

[15] Watson WH, Yang XM, Choi YE, Jones DP, Kehrer JP (2004) Thioredoxin and its role in toxicology. Toxicol Sci 78: 3-14. doi:10.1093/toxsci/kfh050. PubMed: 14691207. 14691207

[16] Song JY, Kim KD, Roe JH (2008) Thiol-independent action of mitochondrial thioredoxin to support the urea cycle of arginine biosynthesis in *Schizosaccharomyces* *pombe* . Eukaryot Cell 7: 2160-2167. doi:10.1128/EC.00106-08. PubMed: 18849471. 18849471 PMC2593194

[17] Day AM, Brown JD, Taylor SR, Rand JD, Morgan BA et al. (2012) Inactivation of a peroxiredoxin by hydrogen peroxide is critical for thioredoxin-mediatemd repair of oxidized proteins and cell survival. Mol Cell 45: 398-408. doi:10.1016/j.molcel.2011.11.027. PubMed: 22245228. 22245228

[18] Song JY, Roe JH (2008) The role and regulation of Trxl, a cytosolic thioredoxin in Schizosaccharomyces pombe. J Microbiol 46: 408-414. doi:10.1007/s12275-008-0076-4. PubMed: 18758731. 18758731

[19] Jara M, Vivancos AP, Calvo IA, Moldón A, Sansó M et al. (2007) The peroxiredoxin Tpx1 is essential as a H_2_O_2_ scavenger during aerobic growth in fission yeast. Mol Biol Cell 18: 2288-2295. doi:10.1091/mbc.E06-11-1039. PubMed: 17409354. 17409354 PMC1877099

[20] Vivancos AP, Castillo EA, Biteau B, Nicot C, Ayté J et al. (2005) A cysteine-sulfinic acid in peroxiredoxin regulates H_2_O_2_-sensing by the antioxidant Pap1 pathway. Proc Natl Acad Sci U S A 102: 8875-8880. doi:10.1073/pnas.0503251102. PubMed: 15956211. 15956211 PMC1157045

[21] McConell GK, Rattigan S, Lee-Young RS, Wadley GD, Merry TL (2012) Skeletal muscle nitric oxide signalling and exercise: a focus on glucose metabolism. Am J Physiol Endocrinol Metab 303: E301-E307. doi:10.1152/ajpendo.00667.2011. PubMed: 22550064. 22550064

[22] Moncada S, Erusalimsky JD (2002) Does nitric oxide modulate mitochondrial energy generation and apoptosis? Nat Rev Mol Cell Biol 3: 214-220. doi:10.1038/nrm762. PubMed: 11994742. 11994742

[23] Hess DT, Matsumoto A, Kim SO, Marshall HE, Stamler JS (2005) Protein S-nitrosylation: Purview and parameters. Nat Rev Mol Cell Biol 6: 150-166. doi:10.1038/nrm1569. PubMed: 15688001. 15688001

[24] Kig C, Temizkan G (2009) Nitric oxide as a signaling molecule in the fission yeast *Schizosaccharomyces* *pombe* . Protoplasma 238: 59-66. doi:10.1007/s00709-009-0074-3. PubMed: 19795185. 19795185

[25] Cao BJ, Reith MEA (2002) Nitric oxide scavenger carboxy-PTIO potentiates the inhibition of dopamine uptake by nitric oxide donors. Eur J Pharmacol 448: 27-30. doi:10.1016/S0014-2999(02)01908-8. PubMed: 12126967. 12126967

[26] Janssen-Heininger YM, Mossman BT, Heintz NH, Forman HJ, Kalyanaraman B et al. (2008) Redox-based regulation of signal transduction: principles, pitfalls, and promises. Free Radic Biol Med 45: 1-17. doi:10.1016/j.freeradbiomed.2008.03.011. PubMed: 18423411. 18423411 PMC2453533

[27] Poole LB, Nelson KJ (2008) Discovering mechanisms of signaling-mediated cysteine oxidation. Curr Opin Chem Biol 12: 18-24. doi:10.1016/j.cbpa.2008.01.021. PubMed: 18282483. 18282483 PMC2408887

[28] Paulsen CE, Carroll KS (2010) Orchestrating redox signaling networks through regulatory cysteine switches. Acs. Chem Biol 5: 47-62. 10.1021/cb900258z PMC4537063 19957967

[29] Benhar M, Forrester MT, Hess DT, Stamler JS (2008) Regulated protein denitrosylation by cytosolic and mitochondrial thioredoxins. Science 320: 1050-1054. doi:10.1126/science.1158265. PubMed: 18497292. 18497292 PMC2754768

[30] Sengupta R, Holmgren A (2012) The role of thioredoxin in the regulation of cellular processes by *S*-nitrosylation. Biochim Biophys Acta 1820: 689-700. doi:10.1016/j.bbagen.2011.08.012. PubMed: 21878369. 21878369

[31] Benhar M, Forrester MT, Stamler JS (2009) Protein denitrosylation: enzymatic mechanisms and cellular functions. Nat Rev Mol Cell Biol 10: 721-732. PubMed: 19738628. 19738628 10.1038/nrm2764

[32] Fourquet S, Huang ME, D'Autreaux B, Toledano MB (2008) The dual functions of thiol-based peroxidases in H_2_O_2_ scavenging and signaling. Antioxid Redox Signal 10: 1565-1576. doi:10.1089/ars.2008.2049. PubMed: 18498222. 18498222

[33] Hess DT, Stamler JS (2012) Regulation by *S*-nitrosylation of protein post-translational modification. J Biol Chem 287: 4411-4418. doi:10.1074/jbc.R111.285742. PubMed: 22147701. 22147701 PMC3281651

[34] Reinartz M, Ding Z, Flögel U, Gödecke A, Schrader J (2008) Nitrosative stress leads to protein glutathiolation, increased *S*-nitrosation, and up-regulation of peroxiredoxins in the heart. J Biol Chem 283: 17440-17449. doi:10.1074/jbc.M800126200. PubMed: 18426799. 18426799

[35] da Silva Dantas A, Patterson MJ, Smith DA, Maccallum DM, Erwig LP et al. (2010) Thioredoxin regulates multiple hydrogen peroxide-induced signaling pathways in Candida *albicans* . Mol Cell Biol 30: 4550-4563. doi:10.1128/MCB.00313-10. PubMed: 20679492. 20679492 PMC2950526

[36] Yamawaki H, Haendeler J, Berk BC (2003) Thioredoxin: a key regulator of cardiovascular homeostasis. Circ Res 93: 1029-1033. doi:10.1161/01.RES.0000102869.39150.23. PubMed: 14645133. 14645133

[37] Aon MA, Stanley BA, Sivakumaran V, Kembro JM, O'Rourke B et al. (2012) Glutathione/thioredoxin systems modulate mitochondrial H_2_O_2_ emission: An experimental-computational study. J Gen Physiol 139: 479-491. doi:10.1085/jgp.201210772. PubMed: 22585969. 22585969 PMC3362521

[38] Takada H, Nishimura M, Asayama Y, Mannse Y, Ishiwata S et al. (2007) Atf1 is a target of the mitogen-activated protein kinase Pmk1 and regulates cell integrity in fission yeast. Mol Cell Biol 18: 4794-4802. doi:10.1091/mbc.E07-03-0282. PubMed: 17881729. PMC2096581 17881729

[39] Koike A, Kato T, Sugiura R, Ma Y, Tabata Y et al. (2012) Genetic screening for regulators of Prz1, a transcriptional factor acting downstream of calcineurin in fission yeast. J Biol Chem 287: 19294-19303. doi:10.1074/jbc.M111.310615. PubMed: 22496451. 22496451 PMC3365961

[40] Veal EA, Findlay VJ, Day AM, Bozonet SM, Evans JM et al. (2004) A 2-Cys peroxiredoxin regulates peroxide-induced oxidation and activation of a stress-activated MAP kinase. Mol Cell 15: 129-139. doi:10.1016/j.molcel.2004.06.021. PubMed: 15225554. 15225554

[41] Kim DU, Hayles J, Kim D, Wood V, Park HO et al. (2010) Analysis of a genome-wide set of gene deletions in the fission yeast *Schizosaccharomyces* *pombe* . Nat Biotechnol 28: 617-623. doi:10.1038/nbt.1628. PubMed: 20473289. 20473289 PMC3962850

