# Supplementary figures and images for: Possible Involvement of Nitric Oxide and Reactive Oxygen Species in Glucose Deprivation-Induced Activation of Transcription Factor Rst2
Source: PLoS One. 2013 Oct 14;8(10):e78012. doi: 10.1371/journal.pone.0078012 (PMC3796501; doi:10.1371/journal.pone.0078012)

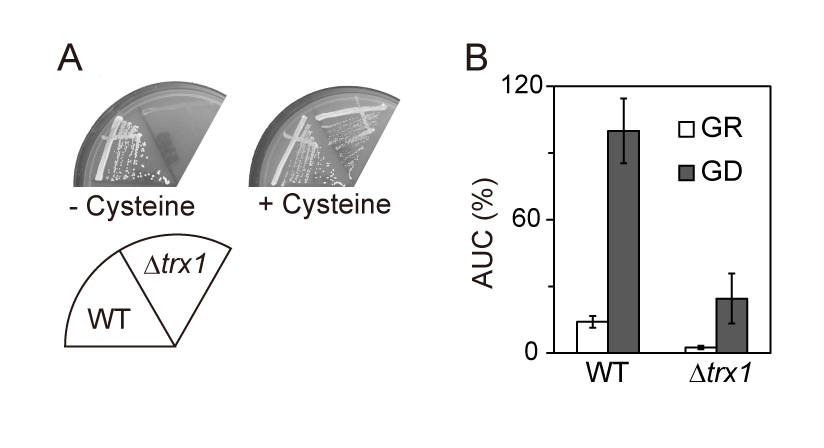

Supplement: Figure S1 — Monitoring of Rst2 transcriptional activity in Δtrx1 cells. (A) Deletion of the trx1+ gene caused cysteine auxotrophy. Wild-type and Δtrx1 cells were streaked onto EMM containing 50 mg/l leucine in the presence (+ Cysteine) or absence of 500 mg/l cysteine (- Cysteine), and cultured at 30°C for 3 days. (B) Monitoring of Rst2 transcriptional activity in Δtrx1 cells. Wild-type and Δtrx1 cells harboring the reporter plasmid were treated with GR and GD in the presence of 500 mg/l cysteine. Error bars, mean ± S.D. (n ≥ 3). (TIF) [file pone.0078012.s001.tif]

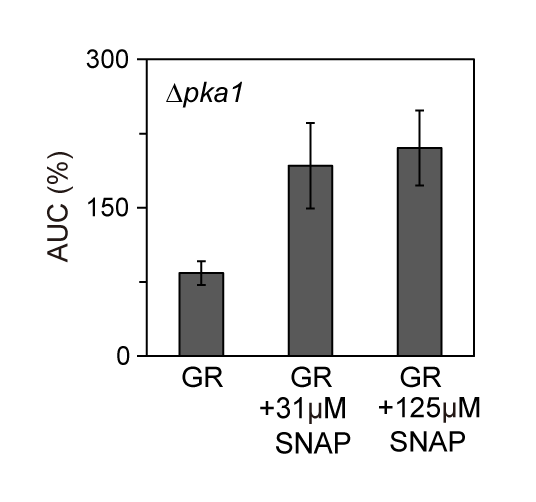

Supplement: Figure S2 — SNAP activated Rst2 transcriptional activity in Δpka1 cells. The Δpka1 cells harboring the reporter plasmid were assayed in GR media in the presence or absence of SNAP. Error bars, mean ± S.D. (n ≥ 3). (TIF) [file pone.0078012.s002.tif]
